# Refrigerated Shelf Life of a Coconut Water-Oatmeal Mix and the Viability of *Lactobacillus Plantarum* Lp 115-400B

**DOI:** 10.3390/foods4030328

**Published:** 2015-08-10

**Authors:** Muthu Dharmasena, Felix Barron, Angela Fraser, Xiuping Jiang

**Affiliations:** Department of Food, Nutrition, and Packaging Sciences, Clemson University, Clemson, SC 29634, USA; E-Mails: muthud@g.clemson.edu (M.D.); afraser@clemson.edu (A.F.); xiuping@clemson.edu (X.J.)

**Keywords:** non-dairy probiotics, coconut water-oatmeal, fermentation, viability, shelf life

## Abstract

Non-dairy probiotic products have the advantage of being lactose-free and can be manufactured to sustain the growth of probiotics. In this study, coconut water and oatmeal were used with the probiotic, *Lactobacillus plantarum* Lp 115-400B (*L. plantarum*) as a starter culture. Two separate treatments were carried out probiotic (P) and probiotic and prebiotic (PP) added. In both treatments, oatmeal-coconut water matrix was inoculated with 7 log CFU/g of *L. plantarum* and fermented at 27 °C for 10 h. For the PP treatment, 1 g of inulin/100 mL of the product was added additionally. The fermented products were then refrigerated (4 °C) and the viability of *L. plantarum*, pH, total acidity, and apparent viscosity of the matrix were monitored at selected time intervals. The shelf life to reach was defined by maintenance of *L. plantarum* count of 7 log CFU/g product. Refrigerated shelf life was determined to be seven-weeks for the P treatment and five-weeks for PP treatment. A significant reduction of pH was observed at the end of the considered shelf life; conversely, the apparent viscosity of the product did not change significantly.

## 1. Introduction

Probiotics have been consumed since pre-historic times as fermented dairy products such as cheese, yogurt, and butter milk. According to the World Health Organization and the Food and Agriculture Organization of the United Nations (WHO/FAO) the definition of probiotics is “live microorganisms, which when administered in adequate amounts, confer a health benefit on the host” [1]. To gain the expected health benefits, “live probiotic bacteria” must be consumed. Some other documented benefits of probiotics consumption include: prevention and reduction of diarrhea, reducing lactose intolerance symptoms [2,3], providing anti mutagenic and anti-carcinogenic properties [3,4], increasing gut mucosal immunity [5,6], decreasing serum cholesterol levels [3,7], and protection from nonalcoholic fatty liver disease (NAFLD) and obesity [8,9]; however, the contribution of bacterial groups in decreasing obesity is unclear as it is always dependent on the heterogeneous nature of microbial genotypes, lifestyle, diet and complex causes of obesity. Though the available evidence of clinical application does not exhibit the direct influences of probiotics and prebiotics on NAFLD and obesity, some studies have already reported less negative effects of those metabolic dysfunctions due to consumption of pre/probiotics [9]. Most of the benefits are obtained by regulating the composition of gut micro biota.

Many authors consider that the number of viable cells required to affect the gastrointestinal environment in humans is between 6–8 log CFU/mL or CFU/g of the food item. This accepted dose is recognized as “the therapeutic minimum” [10,11,12]. Generally, a probiotic product is considered as functional, only if it contains 7 log CFU/mL at the time of consumption [13]. The daily dose of probiotics is considered as 9–11 log CFU [14]. Hence, consumption of 100 mL or g of a product containing the therapeutic minimum (6–8 log CFU/mL or g of the product) would satisfy the daily requirement, although the dose required for health benefits is still being considered. A viable count above 6 log CFU/mL of probiotics and pH above 4.0 of the product were considered as acceptable parameters in determining the shelf life of probiotic products [15]. Nevertheless, some commercial probiotic products may not match the manufacturer claims in the product labels.

Though dairy is the most popular matrix to deliver probiotics to the gastrointestinal tract, a trend of non-dairy probiotics is growing due to various issues with dairy probiotics. Dairy substrates may contain higher cholesterol content and potential allergens such as casein. An alternative to consumers is non-dairy products containing the desired probiotics [16]. This new demand may result in the manufacture of non-dairy probiotic foods such as coconut milk, fruit drinks, nutrition bars, soy products, and cereal based products more and more. Cereal is a worthy food option for fermentation. Previous studies have shown cereal based probiotic products using oat, wheat, malt, and barley with acceptable probiotic live cell counts [13,15,17].

Oatmeal is a well-known breakfast cereal, which is made of oat. Oat fiber is considered as a food source for lactic acid bacteria; the consumed oat fibers serve as colonic food for the intestinal micro biota [18]. In addition, oat contains soluble fibers, which are known to decrease the heart disease by lowering total cholesterol and LDL cholesterol in humans (by 5%–8%) [17,19]. Additional health benefits of the food matrix may lead to production of more consumer-acceptable functional products. Instead of having oatmeal as a regular breakfast meal, it can be consumed as a fermented product with a commercially available and proven probiotic, making for a lifestyle of regular probiotic consumption.

Therefore, the objective of this study was to prepare a novel and safe coconut water-oatmeal based, non-dairy probiotic product, for regular consumption, with a reasonable shelf life under refrigeration storage. Although few studies have been devoted to probiotic survival in non-dairy matrices, to our knowledge, this is the first study where oatmeal and coconut water were tested as a carrier for probiotics.

## 2. Experimental Section

### 2.1. Probiotic Culture

Pure freeze-dried *L. plantarum* Lp 115-400b (*L. plantarum*) strain which has been originally isolated from plant materials, (Danisco, USA) containing approximately 11.60 log CFU/g was used as a starter culture in the study. The strain was stored at −12 °C until use.

### 2.2. Substrate and Substrate Preparation

The substrates used in the experiment were commercially available coconut water (ingredients-young coconut juice, water, sugar, young coconut pulp, sodium metabisulfite as a preservative, (Nakhonpathom, Thailand) and instant plain oatmeal (ingredients-whole grained rolled oats, salt, guar gum, caramel color, vitamins and minerals: Calcium carbonate, Ferric Orthophosphate, Niacinamide, vitamin A palmitate, Pyridoxin hydrochloride, Riboflavin, Thiamin mononitrate, folic acid, and vitamin D, Southern Homes^®^, Jacksonville, FL, USA) for convenience and continuous supply. Oatmeal and pulp free filtered coconut water were sterilized separately at 121 °C for 20 min. The sterile oatmeal was ground in a sterile domestic food processor to produce a fine powder. According to the package instructions, one envelope of oatmeal (28 g) was mixed with a ½ cup of coconut water (125 mL) under sterile ambient conditions. Coconut water and powdered oatmeal were blended together to form a puree. The food matrix was inoculated with *L. plantarum* to have *ca.* 7 log CFU/g. Inulin (Alfa Aesar, MA, USA), a white and odorless powder, was used as a prebiotic in this product preparation. There were two treatments of substrate to be fermented, probiotic added (P) and probiotic and prebiotic added (PP) substrate, and a non-inoculated control (C). All preparations and weighing were conducted aseptically.

### 2.3. Fermentation

Triplicate fermentation batches were prepared for each treatment (C, P, PP) in 250 mL Erlenmeyer flasks, containing 100 mL of oatmeal-coconut water puree. The flasks were fermented at 27 °C for 10 h under static conditions. Then, all experimental flasks were transferred to the refrigerator at 4 °C and stored for 7 weeks.

### 2.4. Shelf-life Analysis

The viability of *L. plantarum* was assessed weekly during the refrigeration storage. The first dilution (10^−1^) was prepared by adding 2 g of the blended mixture with 18 mL of 0.1% peptone water and then 10 fold serial dilutions were prepared. The flasks were returned to refrigerator after sampling. Suitable dilutions were plated in duplicate in MRS agar (EMD Millipore Chemicals, Billerica, MA, USA) and anaerobically incubated using BD gas packs in gas pack jars (BD Diagnostics, Sparks, MD, USA) at 37 °C for 48–72 h.

### 2.5. pH and Acidity Analysis

pH of the matrix (C, P and PP) was monitored on each sampling day. All pH measurements were monitored using a standardized Oakton 510 pH meter (OAKTON Instruments, Vernon Hills, IL, USA) calibrated with standard buffers (pH 7 and 4).

To measure the titratable acidity, 5 g of the product was mixed with 20 mL of water. The titration was performed with standardized 0.1 N NaOH using phenolphthalein as an indicator. Acidity expresses the lactic acid content of the medium. The results were interpreted according to AOAC official methods for fermented dairy products under Section 33.2.06. (1.00 mL of 0.1 N NaOH = 0.0090 g of lactic acid) [20].

### 2.6. Rheological Measurements

Rheological measurements were performed using Brookfield viscometer model HAT (Brookfield; Middleboro, MA, USA). Approximately 150 mL of the sample at 24 °C were used in a 150 mL measuring beaker. Spindle number 3 was used at different speed rotations. The rheological parameters for each sample were measured initially, just after inoculation and at the end of the seven week analysis period as previously described [21].

### 2.7. Statistical Analysis

Analysis of variance was performed for the viability study, pH, and titratable acidity changes using PROC MIXED on the Statistical Analysis System (SAS) version 9.2 (SAS Institute, Cary, NC, USA). The level of significance was set at 0.05. The experiment was performed in triplicate.

## 3. Results and Discussion

### 3.1. Viability Analysis during the Refrigeration Storage of Probiotic Fermented Oatmeal

*L. plantarum* count was enumerated immediately after inoculation to detect the level of initial inoculum and as expected the counts for both treatments were *ca.* 7 log CFU/g product. Under natural conditions using 7 log CFU/mL of viable probiotic cells with cereals provides sufficient growth of probiotic strains and prevents the growth of undesired microorganisms in the cereal matrix [13]. This probiotic strength increases product safety. Our preliminary studies revealed that a fermentation of 10 h was sufficient to carry the cells to stationary phase and the best growth rate was observed at 27 °C. Derzelle *et al.*, also experimentally proved that the optimum growth temperature of *L. plantarum* was 27 °C [22]. Though *L. plantarum* is a facultative hetero-fermentative bacterium, anaerobic conditions and 37 °C were used for incubation simulating the gastrointestinal conditions. The viable counts were raised by 1.5 log CFU/g and 1.0 log CFU/g product after 10 h fermentation for P and PP treatments respectively. The enumerated probiotic population showed an increase of ~2 log CFUs after the first seven days of the refrigeration storage for both treatments. The viability was monitored until the probiotic cell count reached 7 log CFU/g (Table 1).

**Table 1:** The average of probiotic log counts (*n* = 3) of oatmeal coconut water matrix during refrigeration storage (4 °C).

Time (day)	Log CFU/g (P)	Log CFU/g (PP)
0	7.06 ± 0.27_A_^a^	6.99 ± 0.27_A_
7	9.12 ± 0.01_B_	9.01 ± 0.11_B_
14	8.89 ± 0.02_B_	8.75 ± 0.04_B_
21	8.37 ± 0.22_C_	7.97 ± 0.15_C_
28	8.01 ± 0.15_C_	7.46 ± 0.03_D_
35	7.75 ± 0.15_D_	7.05 ± 0.08_A_
42	7.54 ± 0.10_D_	6.66 ± 0.07_E_
49	7.23 ± 0.02_A_	6.41 ± 0.06_E_

^a^ Means within a given column with the same letter are not statistically different from each other (α = 0.05).

The statistical analysis showed that refrigeration storage time and addition of prebiotics have significant effect (*p* < 0.05) on the viability of probiotics. Refrigeration storage time made significant differences in viable counts (*p* < 0.05) between the P and PP samples. Addition of prebiotics did not show a significant effect on the counts compared to the treatment P until day 21 of refrigeration; at this point, growth retardation in inulin added samples was significant (*p* < 0.05).

Studies have shown inconsistency regarding the effect of the inulin on the growth of probiotic bacteria [23,24,25]. Some of them claimed that inulin supported the growth of probiotic bacteria while others contradicted its positive effect. Some studies demonstrated that *L. plantarum* metabolizes prebiotic-related components such as xylo-oligosaccharides in oatmeal, which could be of critical importance for its growth and for potential health benefits caused by the corresponding production of short chain fatty acids in the intestinal mucosa [26].

### 3.2. pH and Acidity Changes of Coconut Water-Oatmeal Matrix

The pH of a food matrix is considered as a critical factor that determines the stability of probiotics during storage [27]. Due to the metabolism of *L. plantarum*, the medium becomes more acidic by decreasing the pH. Probiotics in higher acid matrices are expected to be more tenacious and last longer during the shelf-life of a product. pH of the matrix during fermentation and storage was within the optimum range for the growth of *L. plantarum*, which can grow in a pH range between 3.4 and 8.8 with an optimum value of 6 [28]. During the refrigeration storage treatment P showed slight fluctuations of pH with significant differences (*p* < 0.05). The treatment PP did not show any significant change of pH after fermentation during the refrigeration storage. However, the pH values were slightly lower with the addition of inulin. Interestingly, the control sample showed an increase of pH after 10 h at 27 °C and no significant change during the storage (Table 2).

**Table 2:** The changes of average pH values (*n* = 3) of oatmeal-coconut water matrix during refrigeration storage (4 °C).

Time (day)	pH of P	pH of PP	pH of C
0	5.78 ± 0.00_A_^a^	5.75 ± 0.03_A_	5.79 ± 0.01_A_
7	5.23 ± 0.07_C_	5.08 ± 0.07_B_	6.22 ± 0.03_B_
14	5.18 ± 0.08_C_	5.04 ± 0.17_B_	6.23 ± 0.03_B_
21	5.20 ± 0.13_C_	5.07 ± 0.20_B_	6.24 ± 0.02_B_
28	5.21 ± 0.16_C_	5.08 ± 0.23_B_	6.25 ± 0.02_B_
35	5.22 ± 0.14_C_	5.02 ± 0.27_B_	6.17 ± 0.05_B_
42	5.45 ± 0.11_D_	5.15 ± 0.36_B_	6.21 ± 0.02_B_
49	5.43 ± 0.11_D_	5.12 ± 0.33_B_	6.24 ± 0.01_B_

^a^ Means within a given column with the same letter are not statistically different from each other (α = 0.05).

**Figure 1:**
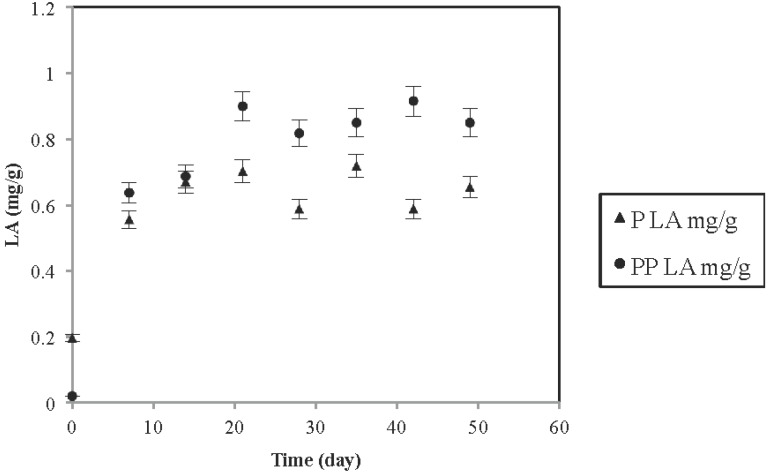
The changes of acidity for fermentation with the effect of prebiotics.

According to the product label, the commercial oatmeal used contained calcium carbonate (CaCO_3_) as a source of calcium, which is used to increase the pH of soil and microbial growth media. Weak organic acids such as lactic acid produced during bacterial fermentation dissociate incompletely resulting in Hydrogen ions (H^+^). These H^+^ ions react with CO_3_^2−^ and form HCO_3_^−^ ions and calcium salt of the weak acid. We assumed that under refrigeration temperatures, fermentation did not progress and the weak organic acids formed during the fermentation got neutralized, increasing the pH of the matrix. However, the pH was above 4, which is recommended for the optimum survivability of *L. plantarum* in a fermented beverage during storage [15,29]. In our study, the drop of the pH was less than one unit (Table 2). The apparent increase in acidity was expressed as mg (lactic acid)/g product and corresponded to the decrease of the pH values showing a higher acidity for inulin added treatment (Figure 1).

### 3.3. Rheological Parameters: Viscosity Analysis

**Figure 2:**
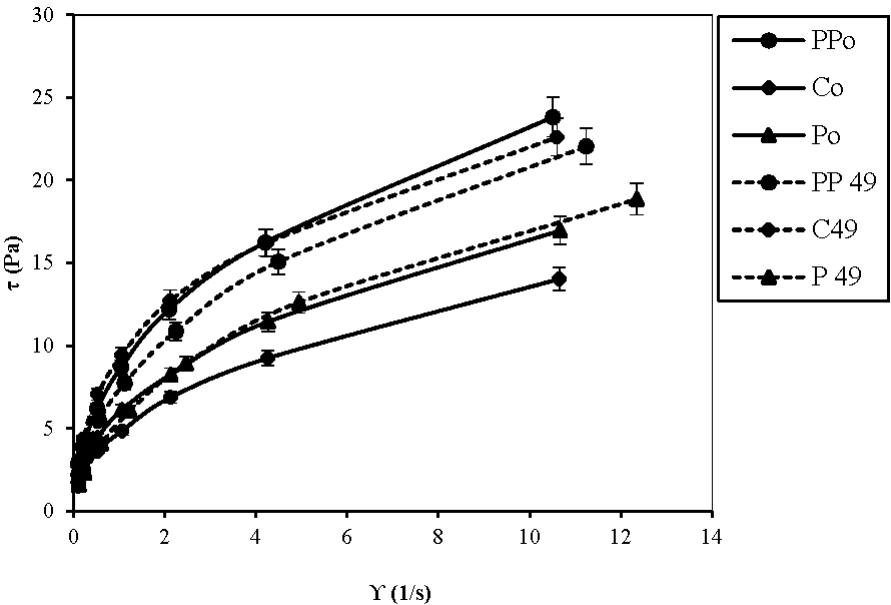
The plots of average shear stress *vs.* average shear rate for three oatmeal-coconut water matrices on the production date (Day 0) and on the expiration date (Day 49).

Some rheological parameters are considered as good indicators of texture and important for the acceptance of the product. In this study, the rheological properties of the product were determined at the beginning and at the end of the refrigerated storage (Figure 2).

The rheological analysis showed that the product behaves as a non-Newtonian fluid with a flow behavior index value of about 0.5 (Table 3).

**Table 3:** The changes of the flow index behavior (n) of oatmeal-coconut water matrix during the shelf life.

Sample	Flow Behavior Index (*n*) on Day 0	Flow Behavior Index (*n*) on Day 49
C	0.47 ± 0.01_A_^a^	0.46 ± 0.04_A_
P	0.46 ± 0.02_A_	0.50 ± 0.06_A_
PP	0.46 ± 0.01_A_	0.51 ± 0.03_A_

^a^ Means within a given row with the same letter are not statistically different from each other (α = 0.05).

**Figure 3:**
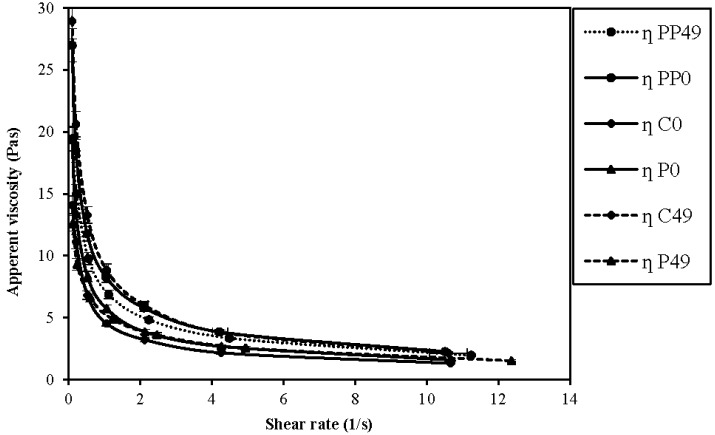
Plots of changes of apparent viscosity with the rising shear rate for the production date and expiration date.

The rheological analysis of the product also showed that the apparent pseudo plastic/shear thinning behavior would not be affected by fermentation or storage of any of the products. Further analysis of the rheological behavior showed that the apparent viscosity decreased with the increasing shearing rate (Figure 3); however, the apparent viscosity did not change significantly for both treatments. This behavior corresponded to the “sheer thinning” effect on the product.

Previous studies have shown that some *L. plantarum* strains (*L. plantarum* EP56) had produced exo-polysaccharide (EPS) [30]. EPS production can be monitored by viscosity measurements [17]. In a previous study of fermenting an oat based non-dairy milk substitute with nine mesophilic LAB strains resulted in final pH of 3.6–5.1. As the proteins in oat do not possess a tendency of coagulating at this pH range, viscosity measurements are appropriate to identify the EPS production. The ideal pH for EPS production is 6.2 [31]. In this study, the samples containing the active probiotics (P and PP) always had a pH > 5.0. Hence, the protein coagulation cannot be expected and any viscosity changes could be due to EPS production by *L. plantarum*. However, the data does not provide a significant change of apparent viscosity.

## 4. Conclusions

This study demonstrated that a refrigerated non-dairy food matrix can support and maintain acceptable levels of viable colony forming units of *L. plantarum* for approximately seven weeks without significant changes in acidity and apparent viscosity. Though the pH change was significant, it was sufficient to determine the shelf life in terms of acidification for a probiotic product. Addition of inulin to the non-dairy food matrix did not improve the probiotic counts and influences the pH in this study. However presence of inulin in the product would be beneficial as a microbial modifier in the intestinal tract. No sensory evaluation was conducted at this time. Further studies are recommended to optimize the concentration and usage of certain prebiotics and sensory evaluation for the consumer acceptance of probiotic products. Our study suggests that home-made coconut water-oatmeal puree is a promising matrix to carry *L. plantarum* in sufficient amounts without changing organoleptic properties of the matrix.
